# Ti_3_C_2_T_x_ MXene-Synergized Intumescent Flame-Retardant Polylactic Acid Composites: Flame Retardancy, Thermal Behavior, and SBS Toughening

**DOI:** 10.3390/polym18172078

**Published:** 2026-08-27

**Authors:** Liqun He, Wan Zhang, Jianji Wang, Jianxiao Bian

**Affiliations:** School of Intelligent Manufacturing, Longdong University, Qingyang 745000, China

**Keywords:** polylactic acid, Ti_3_C_2_T_x_ MXene, intumescent flame retardant, flame retardancy, SBS toughening

## Abstract

Polylactic acid (PLA) is derived from renewable resources and can be composted under controlled industrial conditions, but its flammability and brittleness limit wider use. This study combined Ti_3_C_2_T_x_ MXene with surface terminations and an intumescent flame retardant (IFR) system based on ammonium polyphosphate and pentaerythritol (4:1, *w*/*w*). A styrene–butadiene–styrene (SBS) elastomer was then added to improve toughness. At a total flame retardant loading of 10 wt%, PLA/8.5IFR/1.5MXene reached a limiting oxygen index (LOI) of 30.32% and a UL-94 V-0 rating without dripping. Compared with PLA/10IFR, the peak heat release rate (PHRR) and total heat release (THR) decreased by 18.6% and 28.8%, respectively, and the residue at 600 °C increased from 5.88 to 6.75 wt%. The composite containing MXene formed a denser char and showed a slightly lower Raman D/G intensity ratio. Adding 10 wt% SBS increased notched impact strength by 48% for PLA/IFR and 31% for PLA/IFR/MXene, but reduced tensile strength and modulus. MXene therefore improved the barrier in the condensed phase at a lower IFR content, while SBS partly recovered toughness.

## 1. Introduction

Petroleum-based polymer waste has driven growing interest in renewable and biodegradable alternatives. Polylactic acid (PLA), a bio-based aliphatic polyester derived from renewable resources such as corn starch and sugarcane, has attracted considerable attention for applications in packaging, fibers, biomedical devices, and additive manufacturing owing to its good processability, high transparency, and compostability under controlled industrial conditions [1,2]. Despite these advantages, the widespread use of PLA in fire-sensitive applications is severely limited by its inherent flammability and brittleness [3,4]. Pure PLA typically exhibits a limiting oxygen index (LOI) of 19–21%, a UL-94 rating ranging from V-2 to no rating depending on specimen geometry, and rapid heat release accompanied by severe melt dripping during combustion [5,6]. Moreover, PLA has low impact resistance and elongation at break, which further restrict its structural applications. Therefore, improving the flame retardancy and toughness of PLA without significantly compromising its other desirable properties remains an important research challenge.

Various halogen-free flame-retardant strategies have been developed for PLA, including phosphorus-based additives, intumescent flame-retardant (IFR) systems, bio-based charring agents, silicon-containing compounds, and nanofillers [7,8,9,10]. Among these, IFR systems, which typically combine an acid source (e.g., ammonium polyphosphate, APP), a carbon source (e.g., pentaerythritol, PER), and a gas source, have been widely studied because they can effectively promote the formation of a protective char layer during combustion, thereby reducing heat and mass transfer between the gas phase and the condensed phase [11]. However, conventional IFR systems often require relatively high loadings (typically 15–25 wt%) to achieve satisfactory flame retardancy, which can adversely affect the processability and mechanical strength of the resulting composites. In addition, the char layer formed by IFR alone may be insufficiently robust to withstand the erosive forces of combustion gases, leading to cracks and pores that compromise its barrier effectiveness.

To address these limitations, two-dimensional (2D) nanofillers have been increasingly explored as synergists in IFR-based polymer composites. Layered nanomaterials such as graphene, layered double hydroxides (LDHs), and MXenes can improve flame retardancy by increasing the tortuosity of diffusion pathways for heat, oxygen, and volatile decomposition products, thereby reinforcing the physical barrier effect of the char layer [12,13]. In particular, Ti_3_C_2_T_x_ MXene, a member of the MXene family synthesized by selective etching of the Al layers from the Ti_3_AlC_2_ MAX phase, offers unique advantages over other 2D nanofillers. Unlike graphene, which tends to agglomerate owing to strong van der Waals interactions and has relatively limited surface reactivity in its pristine form, Ti_3_C_2_T_x_ MXene possesses abundant hydrophilic surface terminations (–O, –OH, –F) that facilitate dispersion in polar polymer matrices and promote interfacial interactions. Moreover, the transition-metal (Ti) component of MXene can catalyze char formation and may form Ti-containing oxidation products (e.g., TiO_2_) at elevated temperatures, which can further reinforce the protective char layer. These unique features make Ti_3_C_2_T_x_ MXene a promising synergist for IFR-based PLA composites [14,15]. Nevertheless, the combined effects of surface-terminated MXene and an APP/PER IFR system on the fire performance, thermal stability, crystallization behavior, and mechanical properties of PLA have not been systematically examined, and the underlying structure–property relationships remain incompletely understood.

In addition to flame retardancy, the brittleness of PLA represents another critical limitation that must be addressed for practical applications. Various toughening strategies have been reported, including copolymerization, plasticization, and blending with elastomers or rubbery polymers [16,17,18,19]. Among these, blending with styrene–butadiene–styrene (SBS) block copolymers has been recognized as a practical and cost-effective approach to improve the impact resistance of PLA [20,21]. SBS consists of rigid polystyrene end-blocks and a flexible polybutadiene mid-block, and when dispersed in PLA, the rubbery SBS domains can dissipate deformation energy through cavitation and matrix shear yielding, thereby enhancing toughness. However, the addition of SBS typically reduces the tensile strength and modulus of PLA, and its effects on flame-retardant PLA composites containing IFR and MXene have not been thoroughly investigated.

Recent research has increasingly focused on achieving balanced performance at low additive loadings, including catalytic nanofiller/APP systems [22], recyclable phosphorus–nitrogen zwitterions [23], and chitosan-based multifunctional additives [24]. However, the simultaneous incorporation of MXene as an IFR synergist and SBS as a toughening phase in PLA has not been reported.

Accordingly, this work investigates Ti_3_C_2_T_x_ MXene as a synergist in an APP/PER (4:1, *w*/*w*) IFR system for PLA, with SBS employed as a separate toughening phase. The MXene was synthesized via a LiF/HCl etching route and characterized by Fourier-transform infrared (FTIR) spectroscopy and X-ray diffraction (XRD). The composites were prepared by melt compounding, and their morphology, flame-retardant performance (LOI, UL-94, and cone calorimetry), thermal stability, crystallization behavior, char residue characteristics (SEM and Raman spectroscopy), and mechanical properties were systematically evaluated. The effects of MXene loading and SBS addition on the structure–property relationships and performance trade-offs are discussed, with the aim of providing guidance for the design of flame-retardant, toughened PLA composites with balanced performance.

## 2. Materials and Methods

### 2.1. Materials

Polylactic acid (PLA, trade name REVODE 190) was supplied by Zhejiang Hisun Biomaterials Co., Ltd. (Taizhou, China). Ammonium polyphosphate (APP, CAS No. 68333-79-9, white powder, degree of polymerization *n* ≥ 1000) and pentaerythritol (PER, white crystalline powder, ≥99% purity) were purchased from Aladdin Reagent Co., Ltd. (Shanghai, China). Ti_3_AlC_2_ MAX-phase powder (99 wt% purity) was obtained from Beike Nano Technology Co., Ltd. (Beijing, China). Lithium fluoride (LiF, analytical grade, ≥99.0 wt%) was supplied by Zhongxin Fluoride Materials Co., Ltd. (Shaoxing, China). Hydrochloric acid (HCl, analytical grade, 36.0–36.8 wt%) was purchased from Jiangsu Hengrui Pharmaceutical Co., Ltd. (Lianyungang, China). Calcium hydroxide (Ca(OH)_2_, analytical grade, ≥95.0 wt%), anhydrous ethanol, and deionized water were used as received. Styrene–butadiene–styrene (SBS, trade name Luprene^®^ LG501S) was purchased from LG Chem (Tianjin, China). According to the supplier’s datasheet, LG501S is a linear, non-oil-extended SBS with a styrene content of 31 wt% (S/B ratio = 31/69), a density of 0.94 g/cm^3^, and a Shore A hardness of 78–79.

### 2.2. Preparation of Ti_3_C_2_T_x_ MXene Nanosheets

First, the etching solution was prepared by adding 4 g of lithium fluoride (LiF) and 80 mL of 9 mol L^−1^ hydrochloric acid (HCl) into a polytetrafluoroethylene (PTFE) beaker, followed by stirring at 400 rpm for 30 min. Subsequently, 4 g of Ti_3_AlC_2_ (MAX phase) powder was slowly added to the etching solution, and the mixture was continuously stirred at 40 °C for 48 h. After etching, a separate container containing calcium hydroxide was prepared for the safe treatment of fluoride-containing acidic waste. The resulting reaction mixture was evenly divided into six centrifuge tubes and washed with deionized water by centrifugation at 3500 rpm for 5 min. After each centrifugation cycle, the supernatant was discarded and fresh deionized water was added. This washing procedure was repeated until the pH of the supernatant exceeded 6 [25,26].

After washing, absolute ethanol was added to each centrifuge tube, and the samples were ultrasonicated in an ice bath for 2 h. The resulting suspension was then centrifuged at 10,000 rpm for 10 min, after which the supernatant was removed. Deionized water was subsequently added to the sediment, followed by ultrasonication for 20 min to promote the delamination of the MXene layers. Finally, the suspension was centrifuged at 3500 rpm for 10 min, and the supernatant containing delaminated single-layer Ti_3_C_2_T_x_ MXene nanosheets was collected. The obtained MXene suspension was frozen for 24 h and subsequently freeze-dried for 72 h to remove the solvent, yielding dry Ti_3_C_2_T_x_ MXene powder for subsequent melt blending with PLA.

### 2.3. Preparation of PLA Composites

Before melt compounding, PLA, APP, and PER were vacuum-dried at 80 °C for 24 h. SBS was dried at 60 °C for 8 h. APP and PER were premixed at a mass ratio of 4:1 to form the IFR component.

The composites were prepared in a torque rheometer (PLASTI-CORDER, Brabender, Duisburg, Germany) at 180 °C and 40 rpm. Mixing continued for 8 min after the torque stabilized. In the MXene series, the total IFR/MXene content was fixed at 10 wt%, and MXene replaced part of the IFR. The toughened formulations contained 10 wt% SBS. Table 1 lists the nominal compositions.

After melt blending, the materials were dried at 80 °C for 12 h and hot-pressed into sheets with an LP-S-50 flat vulcanizing machine (Labtech, Fjärås, Sweden). Test specimens were cut to the required dimensions with an RFL-C120000 laser cutting machine (Raycus, Wuhan, China).

### 2.4. Characterization

#### 2.4.1. Morphological Analysis

Composite surfaces, cryogenic fracture surfaces, and cone-calorimetry residues were examined by field-emission scanning electron microscopy (FE-SEM; SU8600, Hitachi, Tokyo, Japan) at 5 kV. Samples were sputter-coated with gold before imaging. Energy-dispersive X-ray spectroscopy (EDS) mapped P and Ti as markers for IFR and MXene, respectively.

#### 2.4.2. Fourier-Transform Infrared Spectroscopy

Fourier-transform infrared (FTIR) spectroscopy was performed with a Nicolet iS50 spectrometer (Thermo Fisher Scientific, Waltham, MA, USA) using the KBr pellet method. The as-prepared Ti_3_C_2_T_x_ MXene powder was mixed with dried KBr at a mass ratio of approximately 1:100 and pressed into a transparent pellet. The spectra were collected in the wavenumber range of 4000–400 cm^−1^ with a resolution of 4 cm^−1^ and 32 scans per sample to characterize the surface functional groups of the MXene.

#### 2.4.3. X-Ray Diffraction

X-ray diffraction (XRD) patterns were recorded with a D8 Advance diffractometer (Bruker, Karlsruhe, Germany) using Cu Kα radiation (λ = 0.154 nm) at 40 kV and 40 mA. The samples were scanned in the 2θ range of 5–80° with a step size of 0.02° and a scanning rate of 5°/min. Both the pristine Ti_3_AlC_2_ MAX-phase powder and the etched Ti_3_C_2_T_x_ MXene powder were analyzed to confirm the removal of Al layers and the expansion of interlayer spacing upon etching.

#### 2.4.4. Limiting Oxygen Index

LOI measurements were performed according to GB/T 2406.2-2009 (equivalent to ISO 4589-2) using the up-and-down procedure [27]. For each formulation, five specimens were tested to determine the critical oxygen concentration, and a single LOI value is reported per formulation, consistent with the standard test method. Specimens measured 100 mm × 6.5 mm × 3 mm.

#### 2.4.5. UL-94 Vertical Burning

Vertical burning tests were performed with a CFZ-3 apparatus (Jiangning Analytical Instrument, Nanjing, China) according to GB/T 2408-2008 [28]. Specimens measured 100 mm × 13 mm × 3 mm and received two 10 s flame applications. Afterflame behavior, dripping, and the UL-94 classification were recorded.

#### 2.4.6. Cone Calorimetry

Cone calorimetry was performed with a TCC918 instrument (Netzsch, Selb, Germany) according to ISO 5660-1:2015 [29]. Specimens measured 100 mm × 100 mm × 4 mm and were exposed to 35 kW/m^2^. Time to ignition (TTI), peak heat release rate (PHRR), time to PHRR (TP), and total heat release (THR) were recorded. Cone calorimetry measurements were performed once per sample, and the results presented are representative single-run data.

#### 2.4.7. Raman Spectroscopy

Raman spectra of the cone-calorimetry residues were collected with a LabRAM Aramis confocal Raman spectrometer (Horiba Jobin Yvon, Palaiseau, France) using 532 nm excitation. Carbon ordering was assessed from the D/G band intensity ratio.

#### 2.4.8. Thermogravimetric Analysis

Thermogravimetric analysis (TGA) was performed with a TG209F1 analyzer (Netzsch, Selb, Germany) under nitrogen at 40 mL/min. Samples were heated from 30 to 600 °C at 10 °C/min. The 5% mass-loss temperature (*T*_onset_), the temperature of maximum mass-loss rate (*T*_max_), and the residue at 600 °C were determined. TGA measurements were performed once per sample under nitrogen atmosphere, and the results presented are representative single-run data.

#### 2.4.9. Differential Scanning Calorimetry

Differential scanning calorimetry (DSC) was performed with a DSC 214 Polymer instrument (Netzsch, Selb, Germany) under nitrogen at 40 mL/min. Samples (5–8 mg) were heated from 0 to 180 °C at 5 °C/min and held for 3 min to remove thermal history. They were then cooled to 0 °C and reheated to 180 °C at the same rate. PLA crystallinity was calculated as follows:

(1) $$\chi_{c}=\frac{\Delta H_{m}-\Delta H_{cc}}{\omega_{f}\Delta H_{m}^{0}}$$

where $\Delta H_{m}$ and $\Delta H_{cc}$ are the melting and cold-crystallization enthalpies, respectively; $\omega_{f}$ is the PLA mass fraction; and $\Delta H_{m}^{0}$ is the melting enthalpy of fully crystalline PLA (93 J/g).

DSC measurements were performed once per sample, and the reported thermal parameters are derived from a single heating–cooling–heating cycle.

#### 2.4.10. Mechanical Properties

Tensile tests were performed with an Instron 5960 universal testing machine (Instron, Norwood, MA, USA) according to GB/T 1040-2006 [30] at 5 mm/min. The specimens were 2 mm thick and had a 20 mm gauge length. They were dried at 70 °C for 12 h before testing. Notched Izod impact tests were performed with a CEAST 9050 pendulum impact tester (Instron, Norwood, MA, USA) (5 J) according to GB/T 1043-2018 [31]. Impact specimens measured 80 mm × 10 mm × 2 mm and had a 2 mm notch. Five specimens were tested for each formulation.

## 3. Results

### 3.1. Characterization of Ti_3_C_2_T_x_ MXene

**Figure 1:**
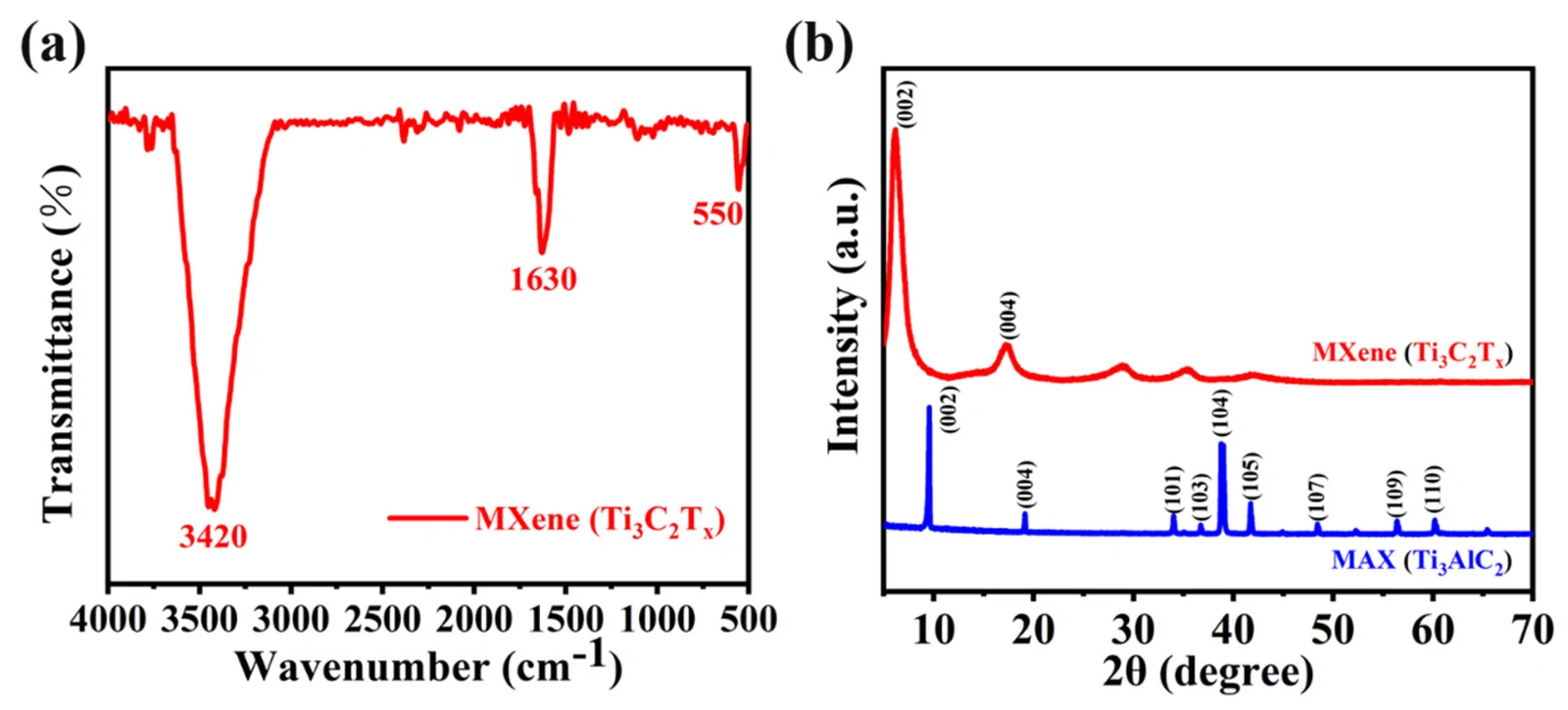
(**a**) FTIR spectra of MXene. (**b**) XRD pattern of MXene.

The chemical structure and surface functional groups of the as-prepared Ti_3_C_2_T_x_ MXene were characterized by FTIR spectroscopy, as shown in Figure 1a. The broad absorption band centered at 3420 cm^−1^ is attributed to the stretching vibration of O–H bonds, arising from adsorbed water molecules and hydroxyl (–OH) terminations on the MXene surface. The band at 1630 cm^−1^ corresponds to the bending vibration of adsorbed H_2_O (δ H–O–H), further confirming surface hydration. The peak observed at 550 cm^−1^ is assigned to Ti–O/Ti–C vibrations within the MXene framework, indicating the preservation of the layered carbide structure. Collectively, these characteristic bands verify the successful synthesis of surface-functionalized Ti_3_C_2_T_x_ MXene.

The crystal structures of the pristine Ti_3_AlC_2_ MAX phase and the etched Ti_3_C_2_T_x_ MXene were investigated by XRD, and the patterns are presented in Figure 1b. The MAX phase (blue curve) exhibits sharp diffraction peaks indexed to the (103), (104), (105), and (107) planes, consistent with its highly crystalline hexagonal structure. After selective etching of the Al layers, the resulting MXene (red curve) displays well-resolved basal-plane reflections indexed to (002), (004), (006), and (008). Notably, the (002) peak shifts to a lower 2θ angle (approximately 7°), indicating an expansion of the interlayer spacing upon Al removal and intercalation. Weak residual peaks corresponding to the (104), (105), and (107) planes are also detectable, suggesting the presence of a minor fraction of unetched MAX phase. Nevertheless, the dominant and ordered (00L) reflections confirm that the MXene retains high crystallinity and an intact layered structure, in good agreement with the FTIR results.

### 3.2. Morphology and Dispersion

**Figure 2:**
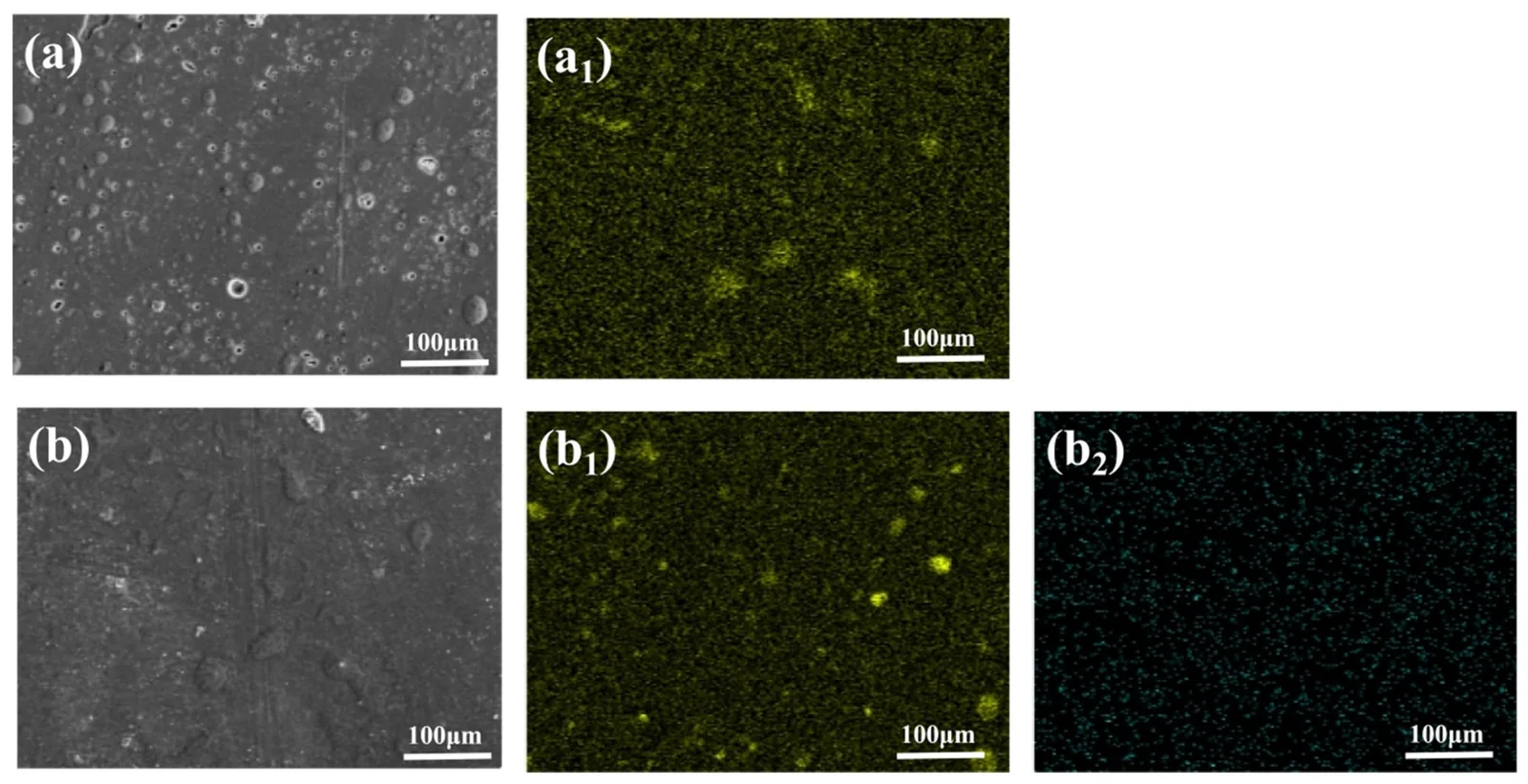
SEM micrographs of composite surfaces and EDS elemental maps: (**a**,**a_1_**) PLA/10IFR; (**b**,**b_1_**,**b_2_**) PLA/8.5IFR/1.5MXene; (**a_1_**,**b_1_**) P maps; and (**b_2_**) Ti map.

As shown in Figure 2a, the PLA/IFR composite exhibits numerous pores on its surface, which can be attributed to the large APP particles in the IFR system. The poor interfacial compatibility between APP and the PLA matrix arises from the mismatch in surface chemistry: APP is a hydrophilic, polar inorganic particle, whereas PLA is a relatively hydrophobic polyester. This difference leads to weak interfacial adhesion between the APP particles and the polymer matrix. During cooling after melt processing, the differential thermal contraction between the inorganic filler and the PLA matrix creates voids and gaps at the particle–matrix interface, resulting in the observed porous morphology.

In contrast, the PLA/IFR/MXene composite (Figure 2b) displays a markedly reduced number of pores and a more homogeneous surface, indicating that the incorporation of functionalized MXene promotes the integration of IFR with the PLA matrix and enhances interfacial compatibility.

To further evaluate the dispersion state of the fillers, EDS elemental mapping was performed. Since the IFR system consists of ammonium polyphosphate (APP) and pentaerythritol (PER), phosphorus (P) was used as a tracer element for IFR, while titanium (Ti), derived from the Ti_3_C_2_T_x_ MXene nanosheets, was employed to identify the distribution of functionalized MXene. As shown in Figure 2(a_1_,b_1_), the P signals are uniformly distributed in both composites, and the Ti mapping (Figure 2(b_2_)) further reveals a homogeneous dispersion of MXene throughout the PLA matrix, with no evident aggregates detected. The uniform distribution of flame-retardant fillers is beneficial for enhancing the flame-retardant performance of the polymer composites. The detailed elemental compositions obtained from the EDS analysis are summarized in Table 2.

### 3.3. Flame-Retardant Performance

#### 3.3.1. LOI and UL-94 Vertical Burning

**Figure 3:**
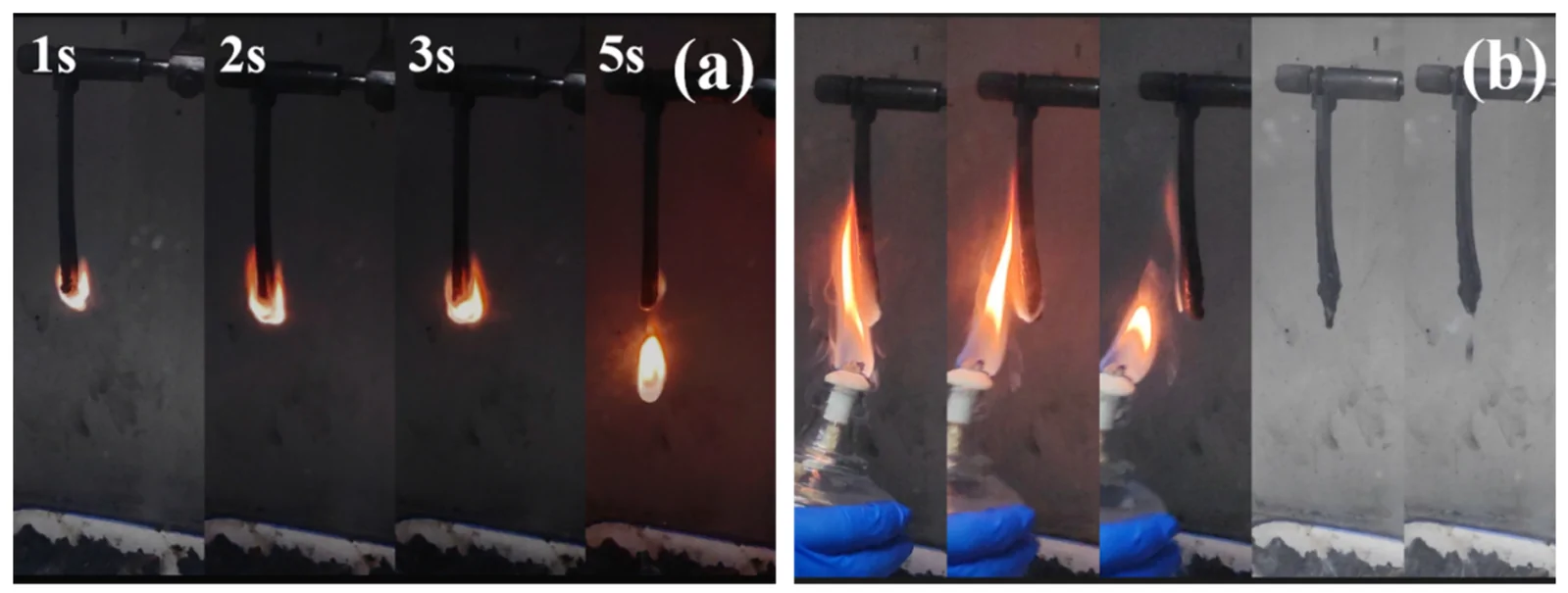
Representative photographs of a PLA/IFR/MXene specimen during the UL-94 vertical-burning test: (**a**) first 10 s flame application; and (**b**) second 10 s flame application.

Table 3 summarizes the LOI and UL-94 results. Figure 3 shows representative images from the vertical-burning test.

Pure PLA had an LOI of 20.00%, showed melt dripping, and received a V-2 rating. Raising the IFR content from 5 to 20 wt% increased the LOI from 28.20% to 32.02%. The first V-0 rating without dripping was obtained at 10 wt% IFR, indicating that this loading is the minimum threshold for effective intumescent flame retardancy in PLA.

At a fixed total IFR/MXene content of 10 wt%, samples with 0.3–0.9 wt% MXene remained V-2 and showed dripping, suggesting that these low MXene loadings cannot fully compensate for the reduced IFR content. Samples with 1.2 or 1.5 wt% MXene reached V-0 without dripping, and PLA/8.5IFR/1.5MXene achieved an LOI of 30.32%, slightly higher than that of PLA/10IFR (29.85%). This MXene loading-dependent trend indicates that Ti_3_C_2_T_x_ actively contributes to flame retardancy, likely through a condensed-phase barrier effect and char reinforcement, rather than acting as an inert filler.

The MXene-containing specimen self-extinguished after both flame applications. Notably, extinction was faster after the second exposure, when a protective residue had already formed on the surface. This observation is consistent with a condensed-phase mechanism, in which the char layer built during the first flame application enhances the barrier effect during subsequent exposure. No sustained dripping was observed for this V-0 formulation.

#### 3.3.2. Cone Calorimetry

**Figure 4:**
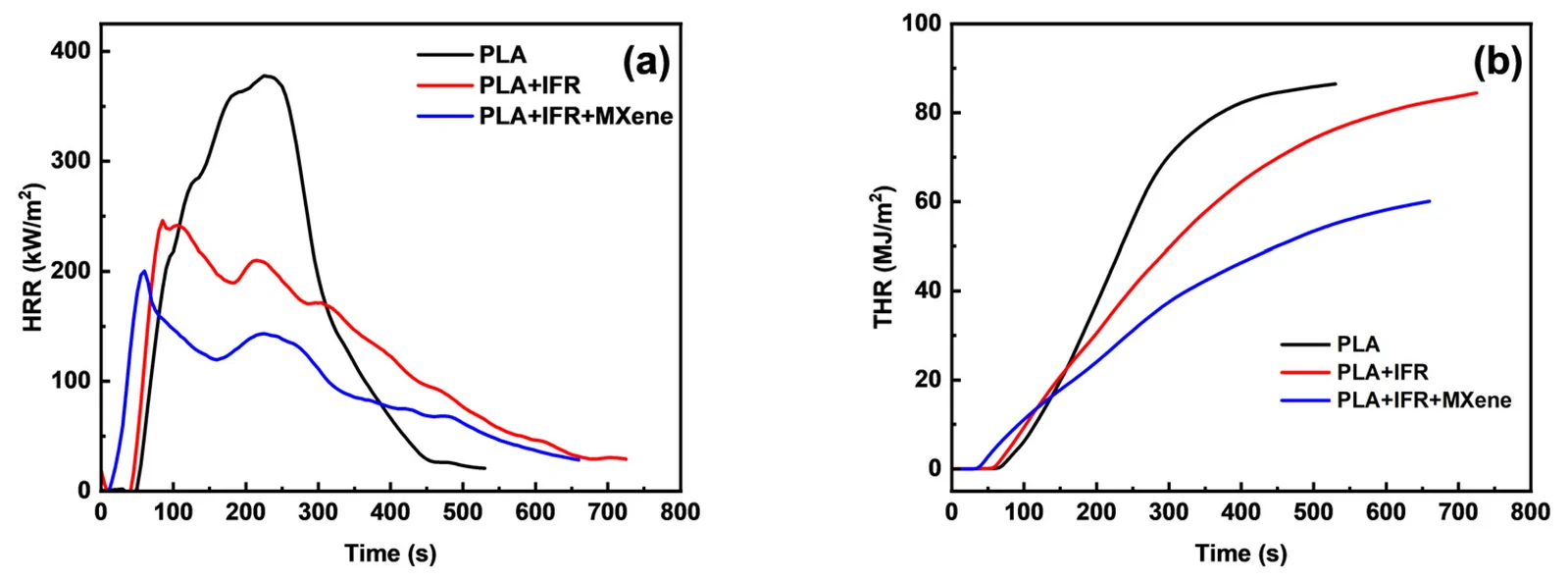
Cone-calorimetry curves of the PLA composites: (**a**) heat release rate; and (**b**) total heat release.

The HRR and THR curves of the PLA composites are shown in Figure 4, and the corresponding parameters are listed in Table 4. Pure PLA exhibited a single sharp HRR peak with a PHRR of 385.82 kW/m^2^ at 215 s, whereas both flame-retardant composites showed broader two-stage HRR curves. The addition of 10 wt% IFR reduced the PHRR to 253.75 kW/m^2^, and replacing 1.5 wt% IFR with MXene further decreased the PHRR to 206.63 kW/m^2^ (18.6% lower than PLA/10IFR), with the TP shortened from 105 to 50 s.

The THR of PLA/8.5IFR/1.5MXene was 60.09 MJ/m^2^, 28.8% lower than that of PLA/10IFR (84.44 MJ/m^2^), indicating enhanced carbon retention in the condensed phase. Notably, the MXene-containing composite ignited earlier (TTI = 38 s vs. 65 s for PLA/10IFR), which is attributed to the high thermal conductivity of MXene accelerating heat transfer and the earlier onset of decomposition. Despite the shorter TTI, the substantial reductions in PHRR and THR confirm that MXene acts primarily through a condensed-phase barrier mechanism.

#### 3.3.3. Char Residue Morphology

**Figure 5:**
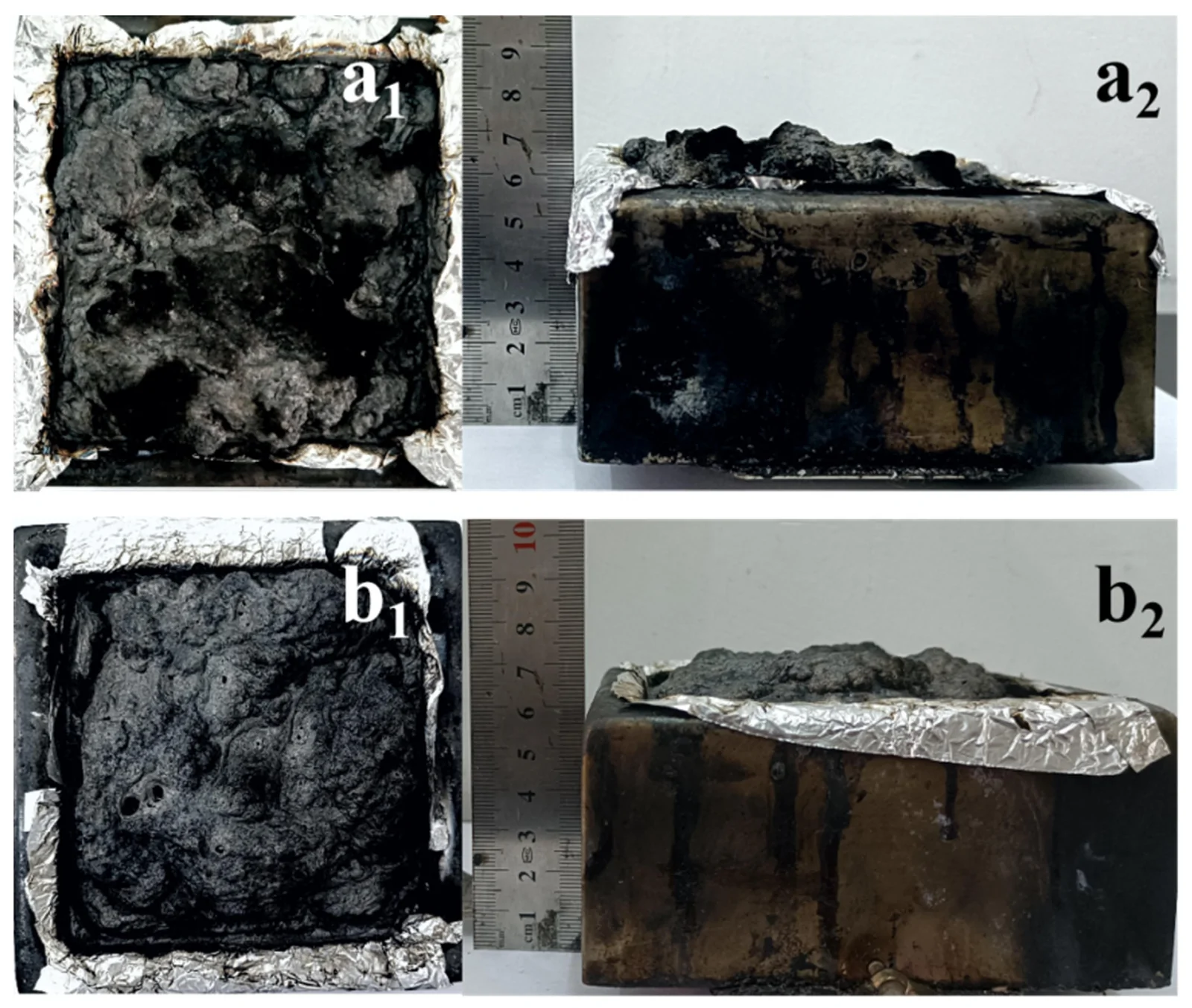
Cone-calorimetry residues: (**a_1_**,**a_2_**) PLA/10IFR; and (**b_1_**,**b_2_**) PLA/8.5IFR/1.5MXene; (**a_1_**,**b_1_**) top views and (**a_2_**,**b_2_**) side views.

The macroscopic morphologies of the cone-calorimetry residues are shown in Figure 5. The PLA/10IFR residue (Figure 5(a_1_,a_2_)) was highly expanded, with a loose, friable structure containing large pores and visible cracks on both the top and side surfaces. These defects can serve as channels for heat and volatile transfer during combustion, compromising the barrier effectiveness of the char. In contrast, the residue of PLA/8.5IFR/1.5MXene (Figure 5(b_1_,b_2_)) exhibited a lower expansion height but a more uniform and compact appearance, with fewer macroscopic cracks and pores. The improved structural integrity of the MXene-containing residue is consistent with its lower PHRR and THR, as a denser char layer provides a more effective physical barrier against heat and mass transfer.

**Figure 6:**
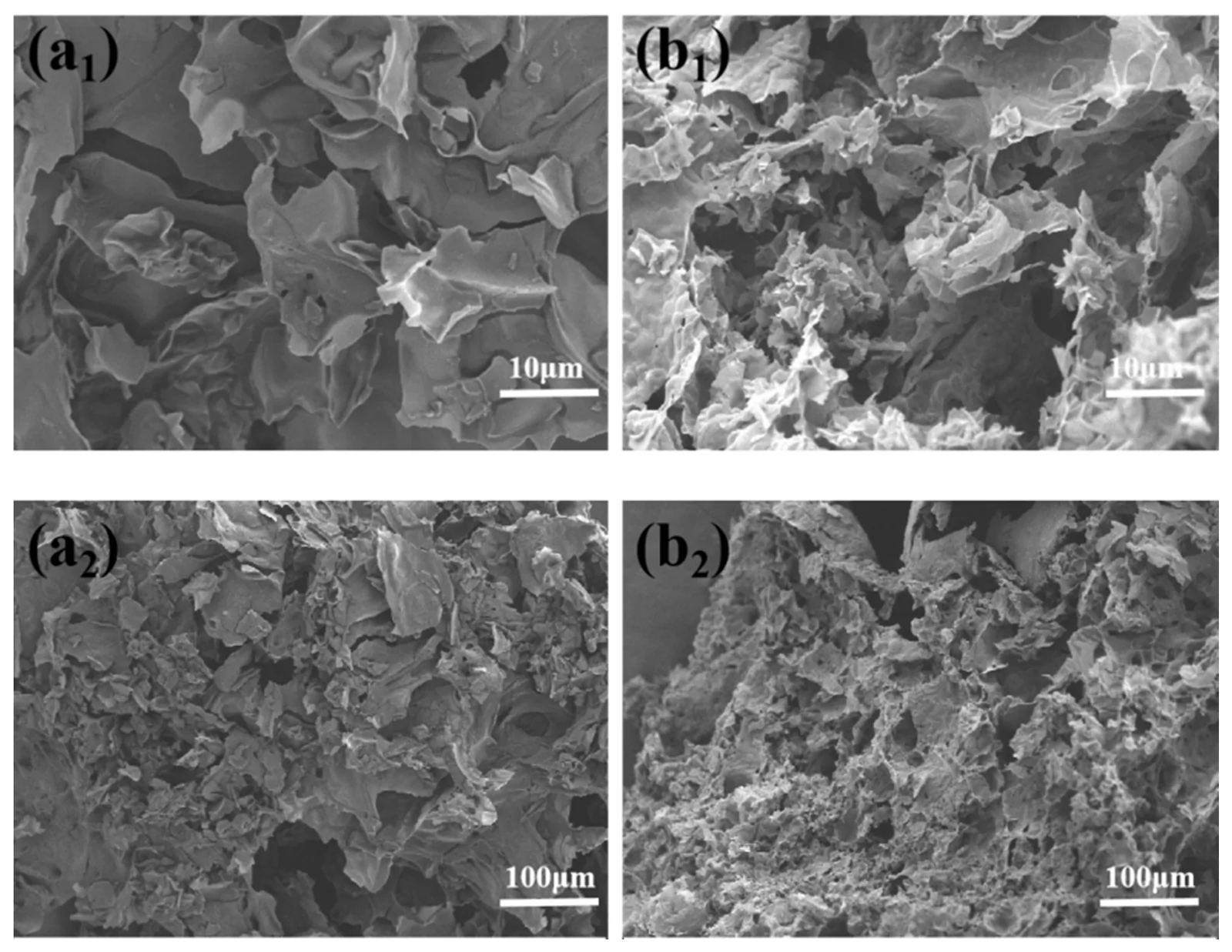
SEM micrographs of cone-calorimetry residues at two magnifications: (**a_1_**,**a_2_**) PLA/10IFR; and (**b_1_**,**b_2_**) PLA/8.5IFR/1.5MXene.

The microscale morphologies of the residues were further examined by SEM, as shown in Figure 6. The PLA/10IFR residue (Figure 6(a_1_,a_2_)) consisted of discontinuous carbonaceous flakes with large, irregular pores, reflecting a weakly consolidated char structure. The PLA/8.5IFR/1.5MXene residue (Figure 6(b_1_,b_2_)), however, displayed a more interconnected and continuous carbonaceous network with substantially smaller pores. This microstructural refinement can be attributed to the 2D Ti_3_C_2_T_x_ nanosheets acting as an inorganic scaffold that promotes the accumulation and consolidation of carbonaceous products during combustion, thereby reinforcing the protective char layer.

#### 3.3.4. Raman Analysis of the Residues

**Figure 7:**
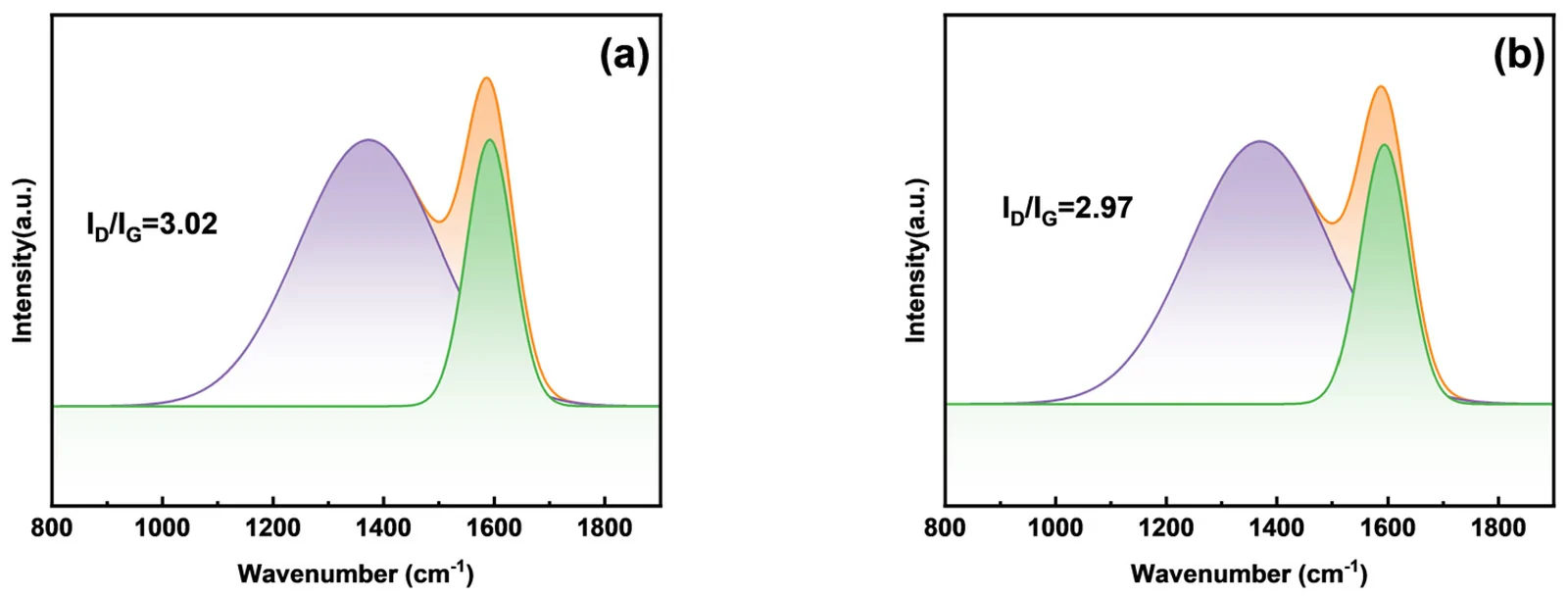
Raman spectra of the cone-calorimetry residues: (**a**) PLA/10IFR (ID/IG = 3.02); and (**b**) PLA/8.5IFR/1.5MXene (ID/IG = 2.97).

To further evaluate the char quality, Raman spectroscopy was performed on the cone calorimetry test (CCT) residues. As shown in Figure 7, both spectra exhibit two prominent peaks: the D band at approximately 1365 cm^−1^, associated with disordered or amorphous carbon, and the G band at approximately 1585 cm^−1^, corresponding to graphitic carbon. The intensity ratio of the D band to the G band (I_D/I_G) is commonly used as an indicator of the carbon ordering of the char residue, where a lower ratio suggests a higher proportion of ordered carbon structures.

The I_D/I_G values were determined to be 3.02 for PLA/IFR and 2.97 for PLA/IFR/MXene. The slightly lower I_D/I_G ratio for the MXene-containing composite may suggest a modest increase in the degree of graphitization of the char layer, which could be attributed to the catalytic effect of MXene. However, it should be noted that the difference between the two samples is small (Δ = 0.05), and the absolute I_D/I_G values may be influenced by the peak-fitting procedure. Therefore, this result should be interpreted as indicative rather than conclusive evidence of a more ordered carbon structure.

### 3.4. Thermal Stability

**Figure 8:**
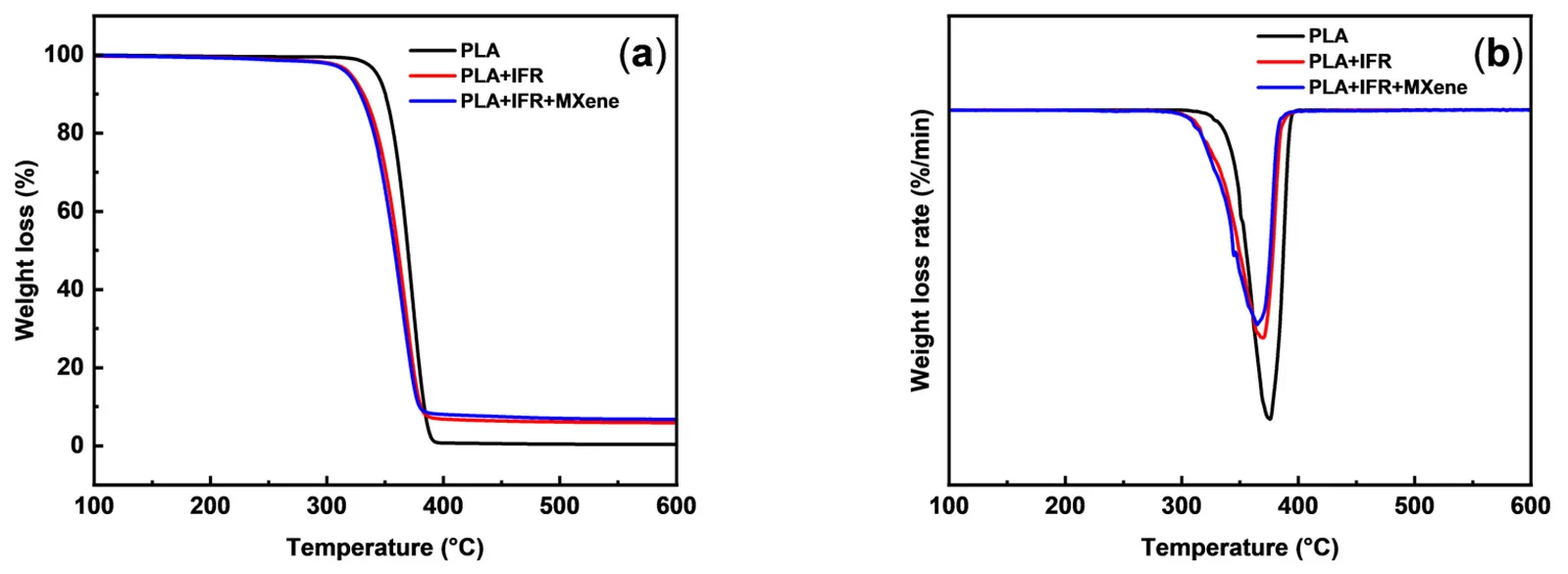
Thermal degradation of the PLA composites under nitrogen: (**a**) TGA curves; and (**b**) DTG curves.

Figure 8 shows the TGA and derivative thermogravimetry (DTG) curves. Table 5 lists the characteristic temperatures and residue yields.

Pure PLA showed mainly one-step mass loss. Its *T*_onset_ and *T*_max_ were 343.22 and 375.82 °C, and the residue at 600 °C was 0.35 wt%. Adding 10 wt% IFR lowered *T*_onset_ to 320.42 °C and *T*_max_ to 369.82 °C. The residue increased to 5.88 wt%, showing that the IFR promoted early dehydration and char formation.

With MXene, *T*_onset_ and *T*_max_ decreased further to 318.61 and 364.61 °C. The residue increased to 6.75 wt%, and the DTG peak became lower. These results point to earlier condensed-phase reactions and greater carbonization during later degradation.

### 3.5. Crystallization Behavior

**Figure 9:**
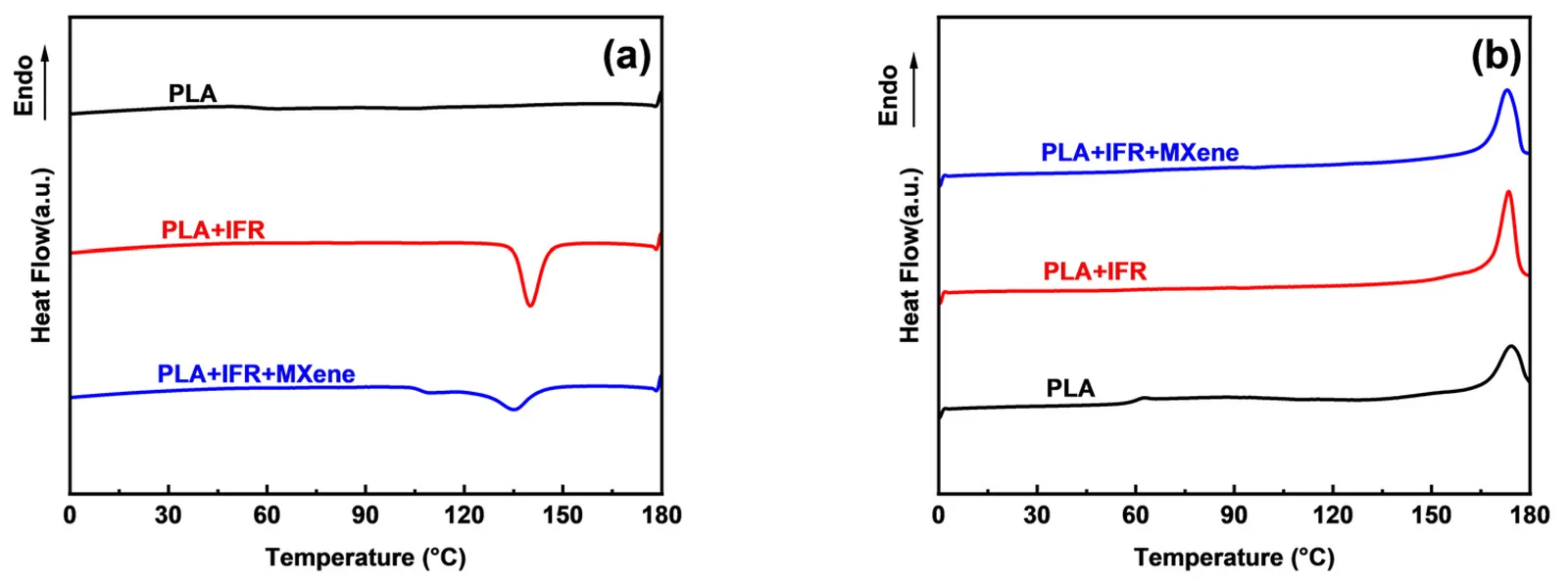
DSC curves of the PLA composites: (**a**) cooling; and (**b**) second heating.

Figure 9 shows the DSC cooling and second-heating curves. The derived parameters are listed in Table 6.

Pure PLA showed no clear crystallization exotherm during cooling at 5 °C/min. During the second heating, PLA/10IFR showed cold crystallization at 140.1 °C and a calculated crystallinity of 3.07%.

For PLA/8.5IFR/1.5MXene, Tcc decreased to 135.1 °C and χc increased to 26.10%. MXene therefore promoted nucleation and reduced the cold crystallization needed during reheating. The shoulder in the second-heating exotherm may arise from overlapping cold crystallization and crystal reorganization. Isothermal kinetics or X-ray diffraction would be needed to confirm this interpretation.

The apparent Tg decreased from 57.1 °C for pure PLA to 43.4 °C for PLA/10IFR, then increased to 52.4 °C after MXene addition. Low-molecular-mass IFR components, interfacial constraints, and crystallinity may all contribute to this trend. Because Tg depends on baseline selection, the interpretation is qualitative.

### 3.6. Mechanical Properties

**Figure 10:**
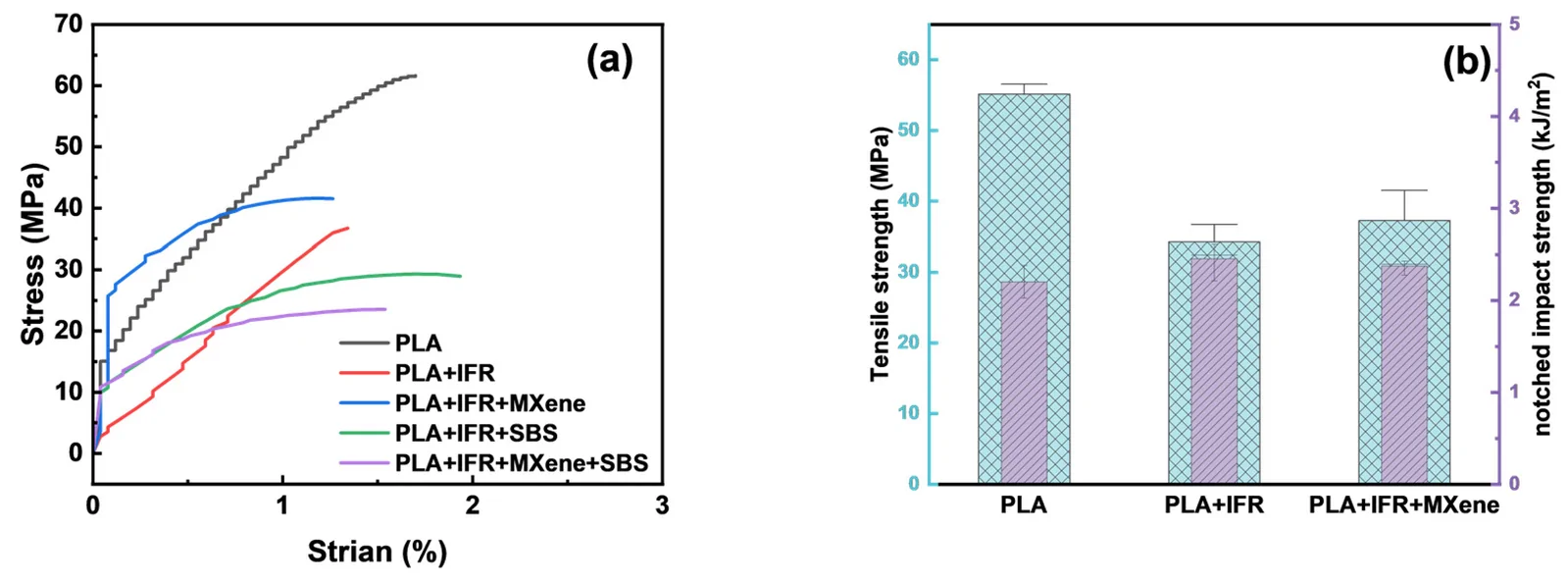
Mechanical properties of the PLA composites: (**a**) tensile stress–strain curves; and (**b**) tensile strength and notched impact strength.

Figure 10 shows the stress–strain curves and the mechanical-property comparison. Table 7 gives the numerical values.

Adding 10 wt% IFR reduced Young’s modulus from 4.64 to 2.79 GPa, tensile strength from 55.14 to 34.22 MPa, and elongation at break from 1.74% to 1.34%. Notched impact strength increased slightly from 2.20 to 2.45 kJ/m^2^.

Replacing 1.5 wt% IFR with MXene partly recovered the modulus and tensile strength to 3.08 GPa and 37.24 MPa. Elongation at break decreased to 1.26%, and impact strength was 2.37 kJ/m^2^. Statistical significance was not assessed, so these numerical differences are treated as trends.

The fracture surface of pure PLA shows typical brittle failure. Voids associated with particles are visible in both composites containing flame retardants. Larger cavities are observed in the sample containing MXene, indicating that a smoother surface does not necessarily correspond to stronger adhesion during mechanical loading.

### 3.7. SBS Toughening

**Figure 11:**
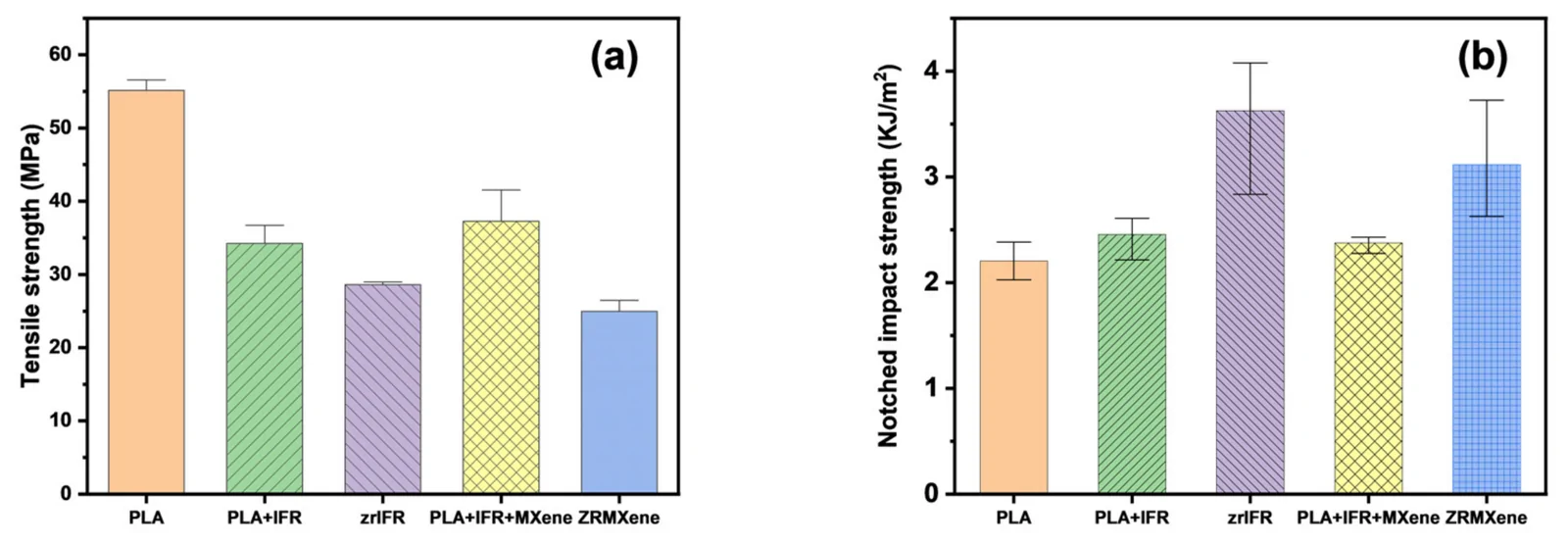
Mechanical properties of SBS-containing PLA composites: (**a**) tensile strength; and (**b**) notched impact strength. zRIFR = PLA/SBS/IFR; zRMXene = PLA/SBS/IFR/MXene.

The formulations containing flame retardants were modified with 10 wt% SBS to improve toughness. Their mechanical properties are compared in Figure 11 and Table 7.

In PLA/IFR, SBS raised notched impact strength from 2.45 to 3.63 kJ/m^2^, an increase of 48%. Elongation at break increased from 1.34% to 1.94%. In PLA/IFR/MXene, impact strength rose from 2.37 to 3.11 kJ/m^2^, and elongation increased from 1.26% to 1.84%.

The increase in toughness was accompanied by lower stiffness and tensile strength. PLA/SBS/IFR had a tensile strength of 28.62 MPa and a modulus of 1.96 GPa. PLA/SBS/IFR/MXene had corresponding values of 24.97 MPa and 1.94 GPa. SBS therefore improved impact resistance but did not restore the mechanical properties of neat PLA.

Cryogenic fracture images show SBS-rich domains of about 300–500 nm dispersed in PLA. The blurred boundaries suggest interfacial interaction, but the material remains a multiphase blend. The morphology is compatible with controlled debonding or cavitation followed by matrix shear yielding.

## 4. Discussion

The improved flame-retardant performance of the PLA/IFR/MXene composite can be understood in terms of a condensed-phase mechanism involving the cooperative action of the APP/PER system and Ti_3_C_2_T_x_ nanosheets. Upon heating, APP decomposes to release phosphoric and polyphosphoric acid species, which catalyze the dehydration and carbonization of PER and the PLA matrix. The incorporation of MXene appears to enhance this process: TGA results show an increase in char residue at 600 °C (from 5.88 to 6.75 wt%), and SEM observation of the cone calorimetry residues confirms a denser, more continuous char layer for the MXene-containing composite. The two-dimensional Ti_3_C_2_T_x_ nanosheets provide a high-aspect-ratio inorganic scaffold that promotes the accumulation and consolidation of carbonaceous products, thereby reinforcing the protective residue.

A second contribution arises from the physical barrier effect of the layered MXene nanosheets. Dispersed within the matrix, these lamellar sheets increase the tortuosity of diffusion pathways for heat, oxygen, and volatile decomposition products. During combustion, MXene becomes integrated into the phosphorus-rich char, yielding an inorganic/organic hybrid residue that is more compact than that formed by APP/PER alone. This reinforced barrier layer restricts heat transfer from the flame to the underlying polymer and limits the release of combustible volatiles, consistent with the simultaneous reductions in PHRR (–18.6%) and THR (–28.8%).

The surface chemistry of Ti_3_C_2_T_x_ may further contribute to char stabilization. The reactive surface terminations (–O, –OH, –F) and the potential formation of Ti-containing oxidation products at elevated temperatures could act in concert with phosphorus-rich species to promote cross-linking and char consolidation. Although the detailed Ti–P chemical interactions were not directly identified in this study, the higher char yield and denser residue morphology are consistent with this interpretation. The slightly lower Raman I_D/I_G ratio (2.97 vs. 3.02) may suggest a modest increase in carbon ordering; however, given the small difference (Δ = 0.05) and the sensitivity of this parameter to the peak-fitting procedure, this result should be regarded as indicative rather than conclusive.

It is noteworthy that the MXene-containing composite exhibited a shorter time to ignition (TTI). This behavior likely reflects the earlier onset of thermal degradation and carbonization in the APP/PER/MXene system, rather than a failure of the flame-retardant mechanism. Indeed, the principal contribution of MXene occurs after decomposition commences, when the rapidly formed condensed-phase barrier restricts further heat and mass transfer. Thus, a reduced TTI can coexist with substantial decreases in PHRR and THR, indicating that the dominant role of MXene is condensed-phase protection rather than ignition suppression.

Regarding mechanical performance, DSC analysis indicated a slight increase in PLA crystallinity upon MXene incorporation, consistent with heterogeneous nucleation by the high-surface-area nanosheets. The increased crystallinity, together with the intrinsic rigidity of Ti_3_C_2_T_x_, may account for the partial recovery of tensile modulus and strength relative to PLA/IFR. Nevertheless, the composite remained brittle, as rigid inorganic fillers cannot effectively dissipate deformation energy. The addition of SBS introduced a separate toughening mechanism via rubber-domain deformation and matrix shear yielding, increasing notched impact strength by 31% for PLA/IFR/MXene, but at the cost of reduced tensile strength and modulus. These results underscore an inherent trade-off: under the current formulation, a fully optimized balance between fire safety, stiffness, and toughness was not achieved, and enhanced interfacial compatibilization among the PLA matrix, IFR, MXene, and SBS represents a clear direction for future work.

## 5. Conclusions

At a total flame retardant loading of 10 wt%, replacing 1.5 wt% of the APP/PER IFR with Ti_3_C_2_T_x_ MXene increased the LOI from 29.85% to 30.32% and retained a UL-94 V-0 rating without dripping. Compared with PLA/10IFR, the PLA/8.5IFR/1.5MXene composite exhibited reductions in PHRR and THR of 18.6% and 28.8%, respectively, and an increase in char residue at 600 °C from 5.88 to 6.75 wt%. SEM observation of the cone calorimetry residues revealed a denser and more continuous char layer for the MXene-containing composite, while Raman spectroscopy showed a slightly lower I_D/I_G ratio (2.97 vs. 3.02), although this small difference may be influenced by the peak-fitting procedure. These observations are consistent with a condensed-phase action of MXene involving barrier effects and char layer reinforcement; however, further investigation would be needed to fully elucidate the underlying mechanism. The shorter time to ignition (TTI) observed for the MXene-containing composite remains a limitation.

DSC analysis indicated a significant increase in crystallinity upon MXene incorporation, and the MXene-containing composite showed partially recovered tensile modulus and strength relative to PLA/IFR, although the material remained brittle. The addition of 10 wt% SBS increased the notched impact strength by 48% for PLA/IFR and 31% for PLA/IFR/MXene, but was accompanied by reductions in tensile strength and modulus in both systems. These results indicate that, under the current formulation, SBS improves toughness at the expense of stiffness and strength; a favorable balance between fire safety and mechanical performance was not fully achieved, and further compatibilization strategies may be required.

## Figures and Tables

**Table 1:** Nominal compositions of the PLA composites.

Sample	PLA (wt%)	IFR (wt%)	MXene (wt%)	SBS (wt%)
Pure PLA	100	0	0	0
PLA/5IFR	95	5	0	0
PLA/10IFR	90	10	0	0
PLA/15IFR	85	15	0	0
PLA/20IFR	80	20	0	0
PLA/9.7IFR/0.3MXene	90	9.7	0.3	0
PLA/9.4IFR/0.6MXene	90	9.4	0.6	0
PLA/9.1IFR/0.9MXene	90	9.1	0.9	0
PLA/8.8IFR/1.2MXene	90	8.8	1.2	0
PLA/8.5IFR/1.5MXene	90	8.5	1.5	0
PLA/SBS/IFR	80	10	0	10
PLA/SBS/IFR/MXene	80	8.5	1.5	10

IFR = APP/PER at 4:1 (*w*/*w*). The PLA fractions in the SBS-containing formulations were calculated by difference from the additive loadings.

**Table 2:** EDS elemental analysis of the PLA/IFR and PLA/IFR/MXene composites.

	PLA + IFR		PLA + IFR + MXene	
Element	wt%	At%	wt%	At%
C	52.82	60.66	52.57	60.21
O	43.99	37.92	45.10	38.77
Si	0.07	0.03	0.12	0.06
P	3.12	1.39	2.00	0.90
Ti	0	0	0.20	0.06
Total	100.00	100.00	100.00	100.00

**Table 3:** LOI values and UL-94 classifications of the PLA composites.

Sample	LOI (%)	Dripping	UL-94 Rating
Pure PLA	20.00	Yes	V-2
PLA/5IFR	28.20	Yes	V-2
PLA/10IFR	29.85	No	V-0
PLA/15IFR	30.94	No	V-0
PLA/20IFR	32.02	No	V-0
PLA/9.7IFR/0.3MXene	27.91	Yes	V-2
PLA/9.4IFR/0.6MXene	28.01	Yes	V-2
PLA/9.1IFR/0.9MXene	28.33	Yes	V-2
PLA/8.8IFR/1.2MXene	29.12	No	V-0
PLA/8.5IFR/1.5MXene	30.32	No	V-0

**Table 4:** Cone-calorimetry parameters of the PLA composites.

Sample	TTI (s)	PHRR (kW/m^2^)	TP (s)	THR (MJ/m^2^)
Pure PLA	70	385.82	215	86.49
PLA/10IFR	65	253.75	105	84.44
PLA/8.5IFR/1.5MXene	38	206.63	50	60.09

**Table 5:** Thermal-degradation parameters of the PLA composites under nitrogen.

Sample	*T*_onset_ (°C)	*T*_max_ (°C)	Residue at 600 °C (wt%)
Pure PLA	343.22	375.82	0.35
PLA/10IFR	320.42	369.82	5.88
PLA/8.5IFR/1.5MXene	318.61	364.61	6.75

**Table 6:** DSC parameters of the PLA composites.

Sample	Tcc (°C)	ΔHcc (J/g)	Tm (°C)	ΔHm (J/g)	Tg (°C)	χc (%)
Pure PLA	–	–	174.3	31.22	57.1	–
PLA/10IFR	140.1	54.47	173.5	57.06	43.4	3.07
PLA/8.5IFR/1.5MXene	135.1	29.46	173.0	51.44	52.4	26.10

**Table 7:** Mechanical properties of the SBS-containing PLA composites.

Sample	Young’s Modulus (GPa)	Tensile Strength (MPa)	Elongation at Break (%)	Notched Impact Strength (kJ/m^2^)
Pure PLA	4.64 ± 0.23	55.14 ± 4.23	1.74 ± 0.08	2.20 ± 0.36
PLA/10IFR	2.79 ± 0.43	34.22 ± 3.45	1.34 ± 0.13	2.45 ± 0.39
PLA/SBS/IFR	1.96 ± 0.27	28.62 ± 1.03	1.94 ± 0.31	3.63 ± 1.24
PLA/8.5IFR/1.5MXene	3.08 ± 0.55	37.24 ± 8.53	1.26 ± 0.22	2.37 ± 0.15
PLA/SBS/IFR/MXene	1.94 ± 0.29	24.97 ± 2.38	1.84 ± 0.32	3.11 ± 1.09

## Data Availability

The data presented in this study are available from the corresponding authors upon reasonable request.

## Abbreviations

APP, ammonium polyphosphate; DSC, differential scanning calorimetry; DTG, derivative thermogravimetry; EDS, energy-dispersive X-ray spectroscopy; IFR, intumescent flame retardant; LOI, limiting oxygen index; PER, pentaerythritol; PHRR, peak heat release rate; PLA, polylactic acid; SBS, styrene-butadiene-styrene; SEM, scanning electron microscopy; TGA, thermogravimetric analysis; THR, total heat release; TP, time to peak heat release rate; TTI, time to ignition.
